# Supplementary material for: An open-source framework for large-scale, flexible evaluation of biomedical text mining systems
Source: J Biomed Discov Collab. 2008 Jan 29;3:1. doi: 10.1186/1747-5333-3-1 (PMC2276192; doi:10.1186/1747-5333-3-1)
Supplement: Additional file 1 — Evaluation platform source code. The additional file contains the software evaluation framework discussed in this paper. The most current release can be downloaded from SourceForge.net [1]. See the accompanying README file for instructions on installation and use. Also included in the distribution is a collection of UIMA wrappers for some commonly used BioNLP tools and annotated corpora. [file 1747-5333-3-1-S1.zip › bionlp_uima_repository-1.0.1/docs/api/src-html/edu/uchsc/ccp/util/nlp/mention/comparison/IdenticalMentionComparator.html]

```
001    package edu.uchsc.ccp.util.nlp.mention.comparison;
002    
003    import edu.uchsc.ccp.util.nlp.mention.Mention;
004    
005    /**
006     * The IdenticalMentionComparator enforces a strict comparison of mention names. The mention names must be identical (although it is not
007     * case-sensitive).
008     * 
009     * @author williamb
010     * 
011     */
012    public abstract class IdenticalMentionComparator extends MentionComparator {
013    
014        /**
015         * Compare the mention names for the two mentions. If they are identical, return true, else return false;
016         */
017        public boolean hasEquivalentMentionNames(Mention mention1, Mention mention2) {
018            return mention1.getMentionName().equalsIgnoreCase(mention2.getMentionName());
019        }
020    
021    }
```
